# The capsid revolution

**DOI:** 10.1093/jmcb/mjad076

**Published:** 2023-11-30

**Authors:** Ian A Taylor, Ariberto Fassati

**Affiliations:** M acromolecular Structure Laboratory, The Francis Crick Institute, London NW1 1AT, UK; Division of Infection and Immunity, University College London, London WC1E 6JF, UK; Institute of Immunity and Transplantation, University College London, London NW3 2PP, UK

**Keywords:** HIV-1 capsid, CPSF6, Lenacapavir, integration, IP6, nucleus, reverse transcription

## Abstract

Lenacapavir, targeting the human immunodeficiency virus type-1 (HIV-1) capsid, is the first-in-class antiretroviral drug recently approved for clinical use. The development of Lenacapavir is attributed to the remarkable progress in our understanding of the capsid protein made during the last few years. Considered little more than a component of the virus shell to be shed early during infection, the capsid has been found to be a key player in the HIV-1 life cycle by interacting with multiple host factors, entering the nucleus, and directing integration. Here, we describe the key advances that led to this ‘capsid revolution’.

## Introduction

Human immunodeficiency virus type-1 (HIV-1), which causes acquired immunodeficiency syndrome (AIDS), was isolated 40 years ago (Barre-Sinoussi et al., 1983), and thus far, the AIDS pandemic has killed an estimated 40 million people. Every year, HIV-1 still infects 1.5 million people and causes 650000 deaths (www.unaids.org). The AIDS pandemic is clearly not over yet, and the lack of an effective vaccine means that it will not be over soon. Nonetheless, significant progress has been made in reducing HIV-1 transmission and mortality from AIDS. Most of this progress can be ascribed to constantly improving antiretroviral drug regimens, which are also effective in pre-exposure prophylaxis (PrEP), although PrEP uptake has been sub-optimal (Bavinton and Grulich, 2021). Combined antiretroviral therapy (cART) reduces viral load to undetectable levels, restores CD4^+^ T cell counts, limits transmission, and extends the life expectancy of people living with HIV-1 to near normal, at least in countries with a good health care system (Lohse and Obel, 2016). cART has contributed and will substantially contribute to the World Health Organization’s goal of ending the AIDS pandemic through the 95/95/95 strategy, whereby by 2030, 95% of people living with HIV-1 are diagnosed, 95% of those diagnosed with AIDS are treated with cART, and 95% of those treated with cART achieve complete viral load suppression (www.unaids.org). However, viral resistance to cART can emerge and spread, especially in settings where access to clinics for regular monitoring is difficult (Chimukangara et al., 2019; Crowell et al., 2021). cART does not cure HIV-1 infection because the virus persists in latent reservoirs. Thus, cART is lifelong, posing its own challenges in an older HIV-1-infected population that will increasingly require additional drug treatment for age-related chronic diseases (Smit et al., 2015). It is therefore important to keep developing new antiretroviral drugs with reduced barriers to resistance, optimal tolerability, and minimal drug–drug interactions.

The HIV-1 capsid protein that forms the capsid shell surrounding the viral genome has emerged as a key element in several early steps of the HIV-1 life cycle, in addition to the well-established assembly and maturation stages (AlBurtamani et al., 2021; Scoca and Di Nunzio, 2021; Muller et al., 2022). The capsid was found in the nucleus of infected cells (Zhou et al., 2011) and it was shown to be a determinant of HIV-1 nuclear import (Yamashita et al., 2007) to affect HIV-1 integration (Dismuke and Aiken, 2006; Vozzolo et al., 2010). A series of elegant discoveries demonstrated that the HIV-1 capsid interacts with several host factors at different stages post-viral entry and such interactions regulate reverse transcription, trafficking, nuclear import, and integration (AlBurtamani et al., 2021; Scoca and Di Nunzio, 2021; Muller et al., 2022). Meanwhile, several small molecules have been developed that antagonise the binding of host factors to the HIV-1 capsid (AlBurtamani et al., 2021). Although most of these molecules have been used as research tools to understand the HIV-1 life cycle, one of them, Lenacapavir, also known by its brand name Sunlenca, was approved in December 2022 by the US Food and Drug Administration as a first-in-class long-acting ART (Segal-Maurer et al., 2022). The remarkable potency and favourable pharmacologic profile of Lenacapavir bodes very well for the clinic, and it is likely that more capsid-targeting ART will reach the approval stage. Here, we shall review the key aspects that led to this ‘capsid revolution’.

## A brief overview of the HIV-1 life cycle

HIV-1 is a lentivirus within the Retroviridae family and as such has a diploid (+strand) ribonucleic acid (RNA) genome of ∼9200 nucleotides, which is reverse-transcribed into a double-stranded deoxyribonucleic acid (DNA) molecule (Knipe et al., 2013). The mature virion is ∼100 nm in diameter and composed of an outer lipid bilayer (‘envelope’) that is acquired by the virus upon budding out of the cellular plasma membrane. Underneath the envelope, there is a matrix layer, which provides the spherical shape typical of HIV-1 and provides an anchoring point for incorporation of the Env glycoproteins that engage the cell receptor (Samal et al., 2022). The viral capsid core sits inside the matrix and encases the viral genome, viral enzymes (reverse transcriptase and integrase), viral nucleocapsid proteins, and the viral protein R (Vpr) (Knipe et al., 2013).

The cell receptor for HIV-1 is CD4, whereas CCR5 and CXCR4 are co-receptors; hence, the virus mainly infects T helper lymphocytes and macrophages (Knipe et al., 2013). Fusion of the viral and cell membranes occurs after a series of ordered conformational changes of the Env protein bound to the CD4 receptor and co-receptors (Chen, 2019). After fusion, the viral core is released into the cytoplasm and trafficked toward the nucleus, where the viral RNA is reverse-transcribed into DNA. The intact or partially disassembled core is transported across the nuclear pores. Once reverse transcription is completed in the nucleus, the core is fully disassembled (‘uncoated’), and the pre-integration complex (PIC), containing integrase, orchestrates integration of the viral DNA into host chromosomes (Dharan and Campbell, 2022; Zhang et al., 2022). To generate a new viral particle, the *gag* and *gag-pol* genes are transcribed from the integrated HIV-1 provirus (Sundquist and Krausslich, 2012). Gag and Gag-Pol are translated as polyproteins, which oligomerise into a lattice that assembles at the plasma membrane into an immature spherical capsid core (Briggs et al., 2009; Bharat et al., 2014). As the virus containing the immature capsid buds out from the cell, the viral enzyme protease cleaves Gag in multiple places to yield mature proteins, including matrix, capsid, nucleocapsid, p6, and two spacer peptides, SP1 and SP2 (Knipe et al., 2013). This produces a dramatic rearrangement of the capsid core in a process called viral maturation. Maturation results in the assembly of ∼200–250 capsid hexamers and 12 pentamers from 2500 capsid monomers to form the mature cone or fullerene-shaped capsid core (Briggs et al., 2009).

## Capsid assemblies and the HIV-1 core

**Figure 1:**
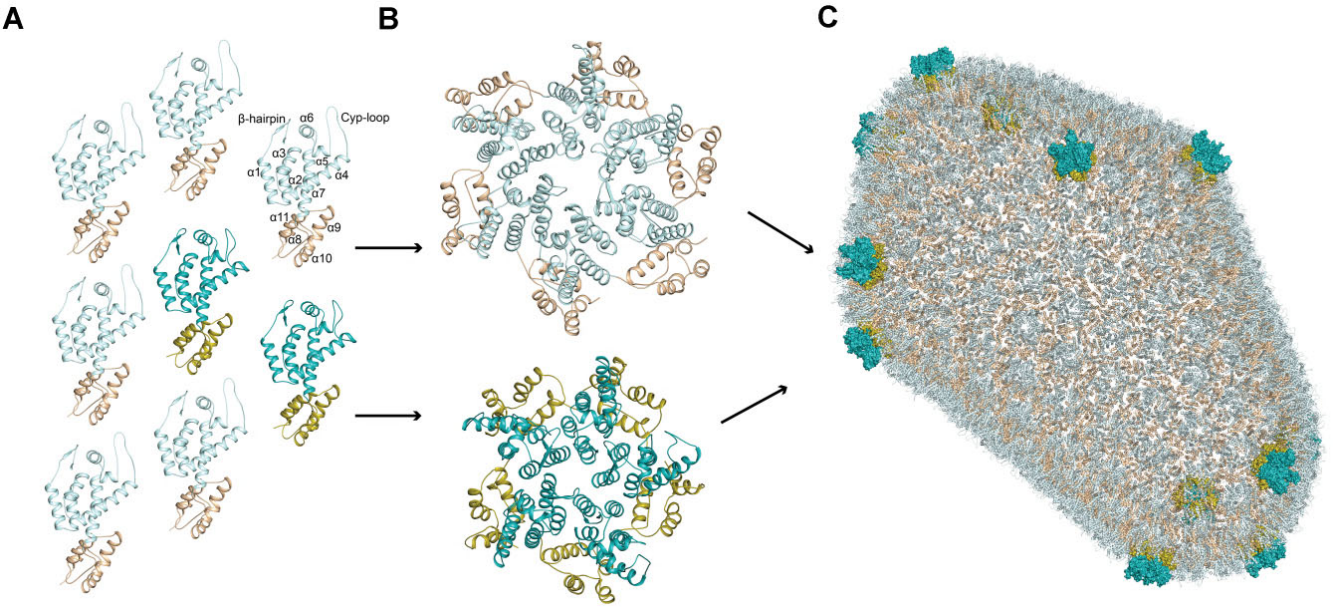
Assembly of the HIV-1 capsid. (**A**) Each capsid monomer is shown in cartoon representation, with CA-NTDs in light or dark cyan and CA-CTDs in wheat or olive. Light and dark shades represent monomers that contribute to capsid hexamers and pentamers, respectively. For illustration, on one monomer, α-helices are labelled sequentially, and the positions of N-terminal β-hairpin and Cyp-loop are indicated. (**B**) Capsid monomers pack into either hexamers (top) or pentamers (bottom). (**C**) The HIV-1 capsid (PDB: 3J3Q). Approximately 200–250 capsid hexamers combine with 12 capsid pentamers to form a closed fullerene cone structure. Hexamers are shown in cartoon representation. The 12 pentamers are distributed toward the ends of the structure and shown in surface representation.

The structure of the HIV-1 capsid has been the subject of intense research effort. Numerous structural studies first defined the capsid monomer, hexamer, and pentamer building blocks (Mortuza et al., 2004; Pornillos et al., 2009, 2011; Gres et al., 2015; Figure 1A and B). Subsequent cryo-electron microscopy (cryoEM), molecular dynamics, and solid-state nuclear magnetic resonance (NMR) studies of whole retroviral cores and *in vitro* assemblies have provided insight into capsid assembly and the 12-pentamer fullerene cone geometry (Ganser et al., 1999) of the capsid lattice at high resolution (Zhao et al., 2013; Mattei et al., 2016; Perilla and Schulten, 2017; Lu et al., 2020; Schirra et al., 2023; Figure 1C). In the assembly, the capsid N-terminal domains (CA-NTDs) form hexamers and pentamers that are displayed on the outer surface of the shell, while the capsid C-terminal domains (CA-CTDs) form a lower layer that is located at dimer and trimer interfaces to assemble the hexamers and pentamers into the closed fullerene cone. The repeating nature of the capsid lattice provides several binding sites for interacting with cellular factors, including (i) the cyclophilin-A (CypA)-binding loop (residues 85–95) that is also bound by nucleoporin 358 (Nup358) (Schaller et al., 2011), (ii) the R18 electropositive pore at the 6-fold axis that is the binding site for deoxynucleoside triphosphates (dNTPs), the host metabolite inositol hexakisphosphate (IP6) (Jacques et al., 2016; Mallery et al., 2018), FEZ1 (Huang et al., 2019), and polyglutamine binding protein 1 (PQBP1) (Yoh et al., 2015, 2022), (iii) the phenylalanine–glycine (FG)-binding pocket surrounded by helices α3–α4–α7 with contribution from adjacent capsid subunits that is the binding site for Sec24C (Rebensburg et al., 2021), CPSF6 (Bhattacharya et al., 2014), and Nup153 (Price et al., 2014), as well as capsid-binding drug molecules, and (iv) the electronegative inter-hexamer junction at the 3-fold symmetry of the capsid lattice that recruits the antiviral protein MxB (Fribourgh et al., 2014; Mallery et al., 2018; Smaga et al., 2019) and the triple arginine motif of Nup153 (Shen et al., 2023).

## Early events post-fusion

**Figure 2:**
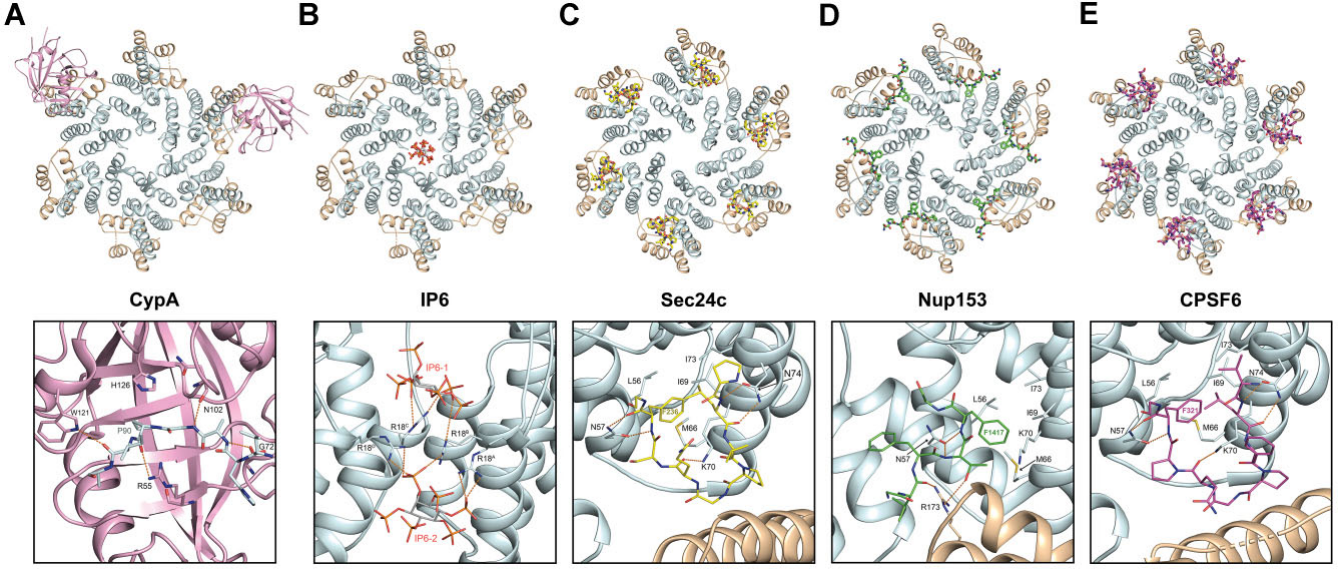
The HIV-1 capsid–natural ligand complexes. The HIV-1 capsid hexamer bound with the cellular factor CypA (PDB: 5FJB; **A**), IP6 (PDB: 6BHT; **B**), Sec24C (PDB: 8CL3; **C**), Nup153 (PDB: 6AYA; **D**), or CPSF6 (PDB: 7ZUD; **E**). In each panel, the protein backbone of the six capsid protomers is shown in cartoon representation, with CA-NTDs in light cyan and CA-CTDs in wheat. (**A**) Upper panel: two molecules of CypA are shown in pink cartoon representation bound to Cyp-loops of non-adjacent capsid protomers within the hexamer. Lower panel: the backbone of CypA is shown in a pink cartoon. Capsid residues in the capsid Cyp-loop that are bound in the active site and CypA residues that make contacts are shown as sticks with hydrogen bonds represented by orange dashed lines. (**B**–**E**) IP6 (**B**), the peptides from Sec24C (**C**), Nup153 (**D**), and CPSF6 (**E**) are shown in stick representation. IP6 is coloured by atom type. Sec24C, Nup153, and CPSF6 are coloured in yellow, green, and magenta, respectively. Lower panels: the capsid residues that interact with bound molecules are labelled and shown in sticks with hydrogen bonds represented by orange dashed lines.

After virus–cell fusion at the cell membrane, the HIV-1 core is released into the cytoplasm, where it binds to host proteins CypA and IP6, although a proportion of CypA and IP6 binds to immature Gag and is delivered along with the virion from producer cells (Luban et al., 1993; Franke et al., 1994; Dick et al., 2018b). CypA binds to the capsid at the so-called Cyp-loop, which is positioned between helices α4 and α5 on the outer surface of the CA-NTD (Figure 1A; Gamble et al., 1996). CypA binds to this loop through a hydrophobic groove on the concave surface of CypA (Figure 2A). This is also the case with the cyclophilin domain of Nup358, albeit the binding is weaker (Bichel et al., 2013). Moreover, the capsid of other lentiviruses that contain Gly–Pro dipeptides in their equivalent Cyp-loops also bind to CypA in an evolutionarily conserved manner. Specifically, in the HIV-1 capsid–CypA complex, residues A88–P93 of the capsid are positioned within the CypA groove where the catalytic residue R55 together with H126 sandwich the G89–P90 peptide bond, facilitating close approach of R55 to catalyse cis–trans isomerisation (Gamble et al., 1996). There are also additional interactions from CypA residues W121, N102, and G72 that further stabilise the interaction. A similar arrangement at the active site is observed in the Nup358–CypA and RELIK–CypA complexes (Goldstone et al., 2010; Bichel et al., 2013). More recently, cryo-EM and solid-state NMR studies have proposed that along with binding at the active site, a secondary site for CypA–capsid interaction can occur with adjacent CA-NTDs in the context of viral cores that can affect capsid stability (Liu et al., 2016), although this issue is controversial (Peng et al., 2019). One study has also implicated the Cyp-loop as the binding site for transportin-1 (Fernandez et al., 2019), although the structural details of the interaction are unknown.

In human cells, CypA was shown to promote the early steps of the HIV-1 life cycle, mainly reverse transcription, suggesting that it binds to the core shortly after fusion (Braaten et al., 1996). The timing of CypA binding to the core has been indirectly measured using the so-called cyclosporin (CsA) washout assay, an *in vitro* assay in which the drug CsA is added at the time of HIV-1 infection to the cells constitutively expressing CypA fused to the restriction factor TRIM5α (TRIMCyp). CsA prevents the interaction between TRIMCyp and the capsid core until it is washed out. In the absence of CsA, TRIMCyp binds to the capsid and causes premature core breakdown, inhibiting reverse transcription, and this readout can be used as a surrogate marker for CypA interacting with the capsid and core uncoating (Hulme and Hope, 2014). Consistent with the notion that CypA promotes reverse transcription, multiple investigators found that TRIMCyp binds to the core during 30—60 min after fusion (Perez-Caballero et al., 2005; Hulme et al., 2011; Chen et al., 2016). CypA tetramers fused to the fluorescent protein DsRed have been used to image the abundance of capsid proteins present in the viral core during the early steps of infection (Francis et al., 2016). These studies revealed that most intracellular viral complexes lost CypDsRed early post-infection, but a few exhibited a slower dissociation that appeared completed at the nuclear pore, suggesting that CypA may remain associated with the viral core until then (Francis et al., 2016; Francis and Melikyan, 2018). At this stage, CypA may be displaced by other host factors, such as Nup358 (Di Nunzio et al., 2012).

The precise mechanism by which CypA aids the early step of the HIV-1 life cycle has remained a mystery for a long time. Recently, CypA was shown to stabilise the capsid core in a concentration-dependent manner in the cytoplasm, which might contribute to the more efficient reverse transcription (Liu et al., 2016). Although *in vitro* CypA is recruited to regions of the capsid with higher curvature (Lau et al., 2020), it has a limited effect on capsid stability (Marquez et al., 2018) other than reducing tube-bundling (Pak et al., 2019). CypA prevents the interaction between human TRIM5α and the viral core in primary CD4^+^ T cells (Kim et al., 2019; Selyutina et al., 2020). This evidence points to indirect effects of CypA on stabilising the viral core and promoting reverse transcription. Furthermore, CypA was recently shown to regulate the pathway for HIV-1 nuclear import. Indeed, POM121 and the FG-rich region of Nup35 bind to the HIV-1 capsid in a CypA-dependent manner, suggesting that CypA modulates specific interactions between the capsid and Nups (Xue et al., 2023). The mechanism for this dependence on CypA is unclear, but it might be related to subtle changes in the curvature of the capsid lattice, which affects host cofactor binding (Lau et al., 2020; Stacey et al., 2023), following CypA binding to the capsid at secondary sites (Liu et al., 2016).

IP6 is essential for virus assembly and maturation. It binds to Lys290 and Lys359 in the CA-CTD and SP1 domains at the centre of the immature Gag hexamer, promoting its formation and subsequent maturation (Dick et al., 2018b). In the mature core, IP6 binds in the central channel of the CA-NTD hexamer (Mallery et al., 2018). Crystal and cryoEM structures show that two IP6 molecules are bound at the 6-fold axis of the hexamer (Dick et al., 2018a, b; Nicastro et al., 2022), respectively sit above and below a basic ring formed by the side chains of Arg18, and make a network of electrostatic interactions with further contributions from a second ring of basic Lys25 side chains (Figure 2B).

In cells, IP6 is retained after virus entry and stabilises the core (Mallery et al., 2018). Biochemical experiments showed that isolated viral cores carry out reverse transcription more efficiently in the presence of IP6 and disassemble with slower kinetics than cores in the absence of IP6 (Mallery et al., 2018). Recent cryoEM evidence showed that reverse transcription carried out *in vitro* in the presence of IP6 can occur in capsid cores of different morphologies, ranging from intact to 50% disassembled (Christensen et al., 2020). Nevertheless, reverse transcription is more efficient in the presence of IP6 than in its absence, indicating that an intact capsid helps the reaction. These results are consistent with studies describing an intact core reaching the nucleus (Muller et al., 2022). CryoEM studies also showed that some capsid cores have lost patches of hexamers and presented DNA loops extruding from the core itself, demonstrating that core disassembly, at least *in vitro*, does not occur in an ‘all-or-none’ fashion but can be partial and/or progressive (Christensen et al., 2020). The possibility of small ruptures of the core is supported by imaging studies using a Gag-GFP fusion protein that is proteolytically cleaved during virus maturation. Some of the cleaved GFP is retained inside the core, and its loss provides a readout for the rupture of the core itself, which appears to occur early in productive infection assays (Mamede et al., 2017, 2021).

Molecular dynamics simulations of intact cores containing two RNA molecules of ∼9500 nucleotides indicated that the addition of IP6 enhances the rigidity of the core, which forms stress-related striations and oscillates at a higher frequency than in the absence of IP6. By increasing the internal pressure and integrating into this model the cryoEM structures previously obtained for cores undergoing reverse transcription, images were obtained of ruptured cores along the main strain lines (Yu et al., 2022). This is consistent with a model whereby IP6 promotes core integrity until the internal pressure, built up by the conversion of ssRNA into dsDNA molecules during reverse transcription, triggers core rupture at regions of greater stress. Similar conclusions were reached using atomic force microscopy to study how reverse transcription affects the core structure *in vitro* (Rankovic et al., 2017, 2021). It is conceivable that DNA synthesis is facilitated in an enclosed container; conversely, the final steps of reverse transcription may be inhibited by a highly confined space, in which dsDNA molecules may not be sufficiently unfolded to allow DNA-dependent DNA synthesis. An important caveat in interpreting these *in vitro* results is that additional host factors bind to the HIV-1 core in infected cells, which may modulate core stability in combination with IP6.

If reverse transcription is one of the triggers for core rupture, the kinetics of DNA synthesis should affect the timing of uncoating. Reverse transcription depends on the availability of dNTPs, and the hexameric central pore that binds with IP6 was also shown to bind with dNTPs and NTPs (Jacques et al., 2016). Hence, the central pore may facilitate reverse transcription by concentrating dNTPs inside the core. Structural studies have indicated that the central pore can alternate between an ‘open’ conformation, which allows the accumulation of dNTPs and NTPs within the core, and a ‘closed’ conformation (Jacques et al., 2016). These two conformations may then influence reverse transcription kinetics, which is important when HIV-1 infects cells that sense cytoplasmic viral DNA, such as macrophages. Rupture of the core and release of viral DNA in the cytoplasm of these cells has been associated with their activation and the establishment of an antiviral state by up-regulation of interferon-stimulated genes (Gao et al., 2013; Rasaiyaah et al., 2013; Yoh et al., 2015; Sumner et al., 2020; Papa et al., 2023). Recently, the predominance of the closed conformation of the hexameric central pore in HIV-1 M relative to HIV-1 O has been linked to the pandemic potential of the M strain and its greater ability to evade sensing in macrophages (Zuliani-Alvarez et al., 2022). Reverse transcription can be initiated and completed in the cytoplasm, from which catalytically active PICs can be extracted (Engelman, 2009; Raghavendra et al., 2010), but DNA synthesis is not a prerequisite for HIV-1 nuclear import (Zaitseva et al., 2009). Recent evidence shows that reverse transcription can be completed in the nucleus (Dharan et al., 2020; Li et al., 2021; Muller et al., 2021; Rensen et al., 2021), which, by delaying core uncoating until shortly before integration, would reduce the risk of DNA sensing in the cytoplasm and perhaps even in the nucleus (Lahaye et al., 2018).

The central pore also binds with PQBP1 (Yoh et al., 2022). PQBP1 interacts with the intracellular DNA sensor cyclic GMP–AMP synthase (cGAS) and recruits it onto the reverse-transcribed HIV-1 DNA and is therefore an important mediator for HIV-1 sensing (Yoh et al., 2015). In monocytes and monocyte-derived dendritic cells, PQBP1 was shown to associate with incoming HIV-1 cores in the cytoplasm. Partially disassembled cores efficiently recruit cGAS in a PQBP1-dependent manner, presumably because viral DNA is presented to cGAS (Yu et al., 2022). At present, it is unclear whether PQBP1 competes with IP6 and dNTPs for binding to the core or whether it stabilizes or de-stabilizes the core itself. In any case, the nature of the PQBP1 interaction with the core and associated host cofactors may turn out to be quite important in understanding the regulation of HIV-1 sensing.

In the cytoplasm, movement of the HIV-1 complex along microtubules is both retrograde and anterograde and depends on dynein and kinesin. The dynein adaptor protein BICD2 (Dharan et al., 2017; Carnes et al., 2018) and the kinesin-1 adaptor protein FEZ1 (Malikov et al., 2015; Huang et al., 2019) bind to the capsid and regulate both HIV-1 capsid transport toward the nucleus and uncoating. FEZ1 contains a negatively charged poly-glutamate region that interacts electrostatically with the highly positively charged R18 residue in the capsid hexamer and possibly other positively charged regions to achieve high avidity (Huang et al., 2019). Therefore, the kinetics of reverse transcription and the trafficking of reverse transcription complexes (RTCs) toward the nucleus may be regulated by the binding of IP6, dNTPs, and FEZ1 on the capsid surface.

## The intermediate to late events post-fusion

Once the viral complex has reached the nuclear membrane, it goes across the nuclear pore complex (NPC) and integrates into host chromosomes. These steps are regulated by several host factors that bind to the capsid at different stages, presumably conferring directionality to the movement. Remarkably, these host factors bind to the same FG-binding pocket in the capsid. This pocket is formed largely by packing of residue side chains displayed on helices α3, α4, and α6 of the NTD with further contribution from the CTD of the adjacent monomer in the capsid hexamer (Figure 2C–E). The common feature of cellular factors that have been shown to bind at the site is an FG dipeptide motif. The host factors include the Sec24C component of the cytoplasmic COPII complex required for viral cytoplasmic trafficking to the nucleus (Rebensburg et al., 2021; Wei et al., 2022; Stacey et al., 2023), Nup153 that is present in the nuclear basket of the NPC (Matreyek et al., 2013; Price et al., 2014), and polyadenylation and splicing factor CPSF6 that is present in the nucleus (Lee et al., 2010, 2012; Price et al., 2012, 2014).

Within the FG-binding pocket, the host factors share a common modality of interaction with the capsid (Figure 2C–E). In each protein, the phenylalanine of the FG motif (F236, F1417, and F321 from Sec24C, Nup153, and CPSF6, respectively) penetrates the capsid FG pocket that is lined with hydrophobic side chains from residues L56, M66, I69, and I73 from the surrounding helices α3 and α4. In addition, the side chain of N57 on α3 forms hydrogen bonding interactions with the main chain around the core phenylalanine to further clamp it into the pocket. This commonality of interaction continues further for the Sec24C- and CPSF6-bound peptides that share nearly identical ‘horseshoe turn’ conformations in the crystal and cryoEM structures (Price et al., 2014; Nicastro et al., 2022; Stacey et al., 2023) and both make hydrogen bonding interactions with the side chains of Lys70 and Asn74 on helix α4. In contrast, the trajectory of the bound Nup153 peptide in the crystal structure follows a different path, and rather than turning back in the horseshoe conformation to interact with residue helix α4, it follows a more linear path, making further hydrogen bonding interactions with the side chain or Arg173 from the CTD of the adjacent capsid protomer (Price et al., 2014). More recently, the mode of binding to the FG pocket by Sec24C, Nup153, and CPSF6 has been shown to be influenced by surrounding low-complexity uncharged and proline-rich sequences, located N- and C-terminal to the FG peptide, that promote binding to the assembled capsid (Wei et al., 2022).

The NPC is an 8-fold symmetric macro-assembly scaffold that spans the nuclear envelope and contains a central channel (Petrovic et al., 2022). This channel is filled with filamentous FG-rich Nups (FG-Nups), which collectively organise into a dynamic hydrogel (Petrovic et al., 2022). This hydrogel forms the selectivity barrier, which is highly hydrophobic and filters out molecules with a diameter >5 nm. Larger molecules need to bind to specialised nuclear transport receptors (NTRs; also called importins or karyopherins) that confer the right hydrophobic properties to the cargos to be ‘chaperoned’ across the channel (Stanley et al., 2017). Transport directionality is mediated by RanGTP that binds to the imported NTR–cargo complex in the nucleus and triggers its dissociation (Stanley et al., 2017). The ability of the HIV-1 capsid to bind to multiple FG domains suggests the intriguing possibility that it may behave as a multimeric NTR, engaging several FG-Nups in the NPC (Di Nunzio et al., 2013; Xue et al., 2023). It is not clear how very large cargoes, with the size approaching the diameter of the central NPC channel, translocate across the nuclear import barrier (Petrovic et al., 2022). Viral cores have been observed translocating the NPC either intact or partially disassembled (Blanco-Rodriguez et al., 2020; Zila et al., 2021), and the central channel can expand its functional diameter up to 65 nm, which facilitates translocation of large cargoes (Petrovic et al., 2022). Furthermore, molecular modelling and atomic force microscopy on the native NPC in physiological buffer indicated that the FG-Nups confined in the central channel behave like polymers with weak intermolecular interactions that can be displaced by NTRs (Osmanovic et al., 2012; Bestembayeva et al., 2015). For most cargoes, the displacement would be small and local, but for a cargo with the size of the HIV-1 capsid, simultaneously engaging many Nups, intermolecular interactions between the FG-Nups would be weakened and replaced by FG-Nup/viral core interactions, inducing a partial collapse of the FG-Nups toward the wall of the central channel (Osmanovic et al., 2012). This ‘bi-stable’ behaviour of the Nups inside the channel, together with the expansion of the channel diameter, may explain how the viral capsid manages to go across the nuclear pore. Furthermore, additional NTRs have been shown to recognise other components of the viral complex, such as integrase (Zaitseva et al., 2009; Ao et al., 2010), and these interactions may promote nuclear import of partially disassembled cores in cooperation with the capsid.

The capsid also affects HIV-1 integration (AlBurtamani et al., 2021). HIV-1 PICs obtained from viruses with hyper-stable capsids showed poor *in vitro* integration activity compared to PICs obtained from wild-type virus (Dismuke and Aiken, 2006). The discovery that the antibiotic Coumermycin-A1 targeted the capsid and inhibited HIV-1 integration further supported this link (Vozzolo et al., 2010; Chen et al., 2016). These pharmacological findings have been recently corroborated by the development of second-generation capsid inhibitors, such as GS-6207 (see also below), which blocks both HIV-1 nuclear import and integration (Link et al., 2020). The mechanism responsible for this phenotype is not known, but it may be related to the need for the PIC to complete uncoating in the nucleus before integration (Zhou et al., 2011; Muller et al., 2021). Transportin-3 was shown to bind to the HIV-1 capsid in a RanGTP-dependent manner and may promote uncoating in the nucleus (Zhou et al., 2011).

CPSF6 is a nuclear protein that functions in processing mRNA for polyadenylation as a component of mammalian cleavage factor 1 (Dettwiler et al., 2004). As described above, CPSF6, Sec24C, and Nup153 recognise the same pocket in the capsid, and certain capsid mutations, such as N74D, abrogate this interaction (Price et al., 2012). By binding to the capsid, CPSF6 directs intranuclear localization of the virus to the actively transcribed chromatin regions, such as nuclear speckles and speckle-associated domains (Francis et al., 2020; Li et al., 2020). Recent studies have shown that if capsid is prevented from binding to CPSF6, PICs localize to the nuclear periphery and integrate more frequently into heterochromatic lamina-associated domains (Achuthan et al., 2018; Francis and Melikyan, 2018) but less frequently into genomic regions enriched for genes involved in T cell activation and metabolism (Zhyvoloup et al., 2017; Chen, 2023). Furthermore, shortly after the PIC enters the nucleus, condensates of CPSF6 form around clusters of RTCs/PICs (Rensen et al., 2021; Scoca et al., 2022). These CPSF6 membraneless organelles have been proposed to be niches where RTCs/PICs mature just before integration (Ay and Di Nunzio, 2023).

## Drug binding at the FG pocket

**Figure 3:**
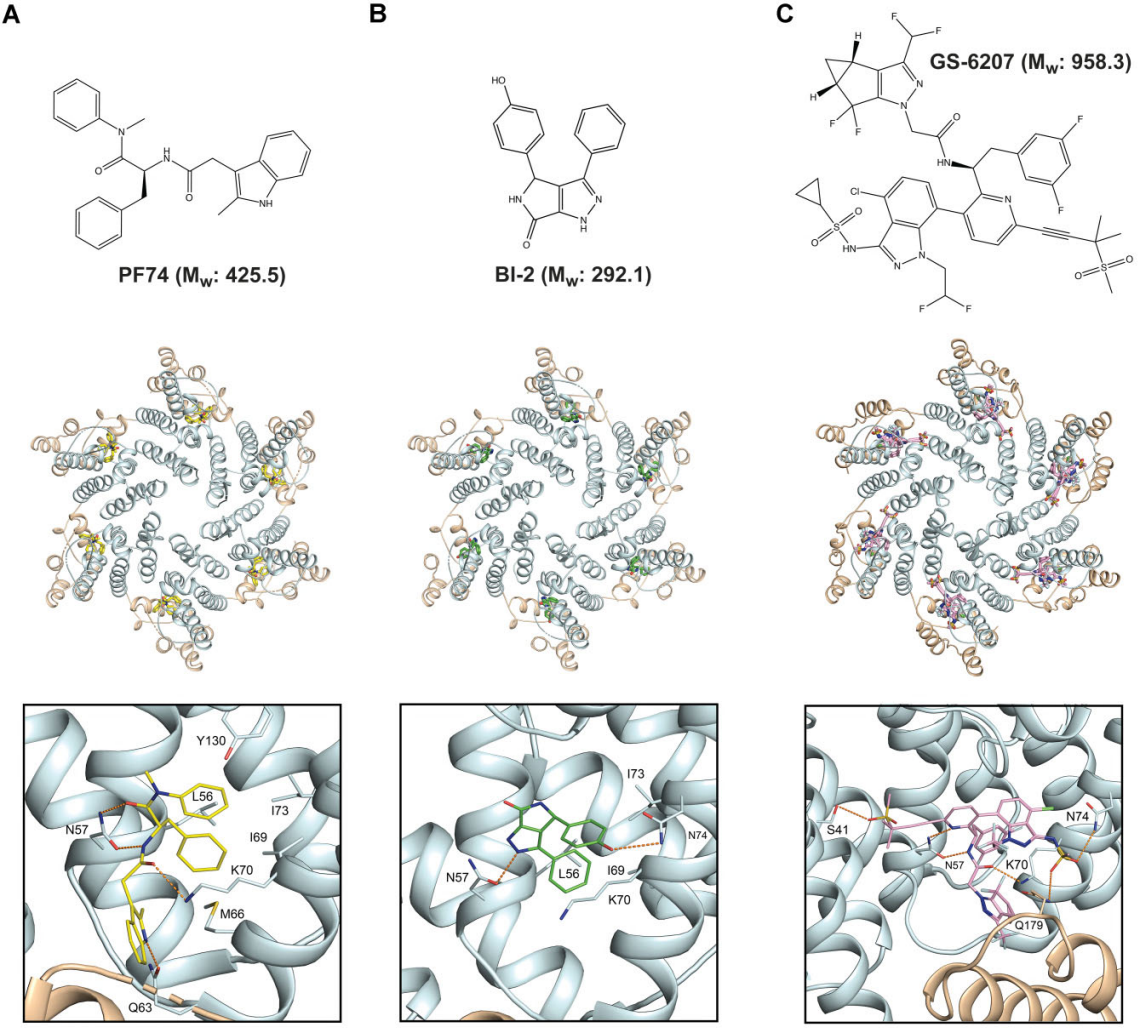
The HIV-1 capsid–drug interactions. Upper panels: chemical structures and relative molecular weights (M_w_) of drug molecules PF74 (**A**), BI-2 (**B**), and GS-6207 (**C**). Middle panels: the HIV-1 capsid hexamer can bind to PF74 (PDB: 4U0E; **A**), BI-2 (PDB: 4U0F; **B**), and GS-6207 (PDB: 6VKV; **C**). The capsid protein backbone is shown in cartoon representation, with CA-NTDs in light cyan and CA-CTDs in wheat. Drugs are shown in stick representation bound at the α3–α4–α7 pocket. Lower panels: details of molecular interactions at the drug binding sites. Drug molecules and capsid residues that make interactions are shown in stick representation. Hydrogen bonds are represented with orange dashed lines.

The FG pocket in capsid is also the binding site for drug compounds that inhibit viral replication through a variety of mechanisms, including capsid destabilisation and stablisation, inhibition of reverse transcription, blocking of nuclear import, and inhibition of integration. The compounds PF-3450074 (PF74), BI-2, Coumermycin-A1, GS-CA1, and GS-6207 (Lenacapavir) all bind at the FG pocket (Blair et al., 2010; Lamorte et al., 2013; Price et al., 2014; Chen et al., 2016; Bester et al., 2020; Link et al., 2020), and crystal and cryoEM structures for the capsid complexes with PF74, BI-2, and Lenacapavir (Figure 3) show how they utilise many of the same interactions as that made by the natural ligands. For instance, the phenyl group of PF74 penetrates the FG pocket and makes equivalent interactions as F321, F1417, and F236 in CPSF6, Nup153, and Sec24C, respectively. The surrounding amide and carbonyl of the PF74 phenyl group also make equivalent hydrogen bonding interactions with the N57 side chain that is observed in the naturally bound peptides. Further hydrogen bonding with the K70 and Q63 side chains completes the complementary drug–capsid interactions (Figure 3A). BI-2 and the related BI-1 also bind in the FG pocket with a phenyl group that packs against the surrounding hydrophobic side chains of L56, L69, and I73 and the aliphatic portion of K70. However, while the interaction with N57 is conserved with PF74, BI-2 does not make further interactions with Q63 and K70 but instead makes hydrogen bonding interactions with the N74 side chain (Figure 3B). It is apparent that there is a cadre of interactions made by the natural ligands and the first-generation drugs in and around the FG-binding pocket. However, of the total number of interactions observed, each compound or natural ligand only utilises one subset. For example, CPSF6 makes hydrogen bonding interactions with N57, K70, and N74. In contrast, Nup153 interacts with N57 but not N74 and makes an additional interaction with R173 from the CTD of the adjacent capsid monomer. Similarly, both PF74 and BI-2 contain a phenyl moiety that sits into the FG pocket and makes hydrogen bonding interactions with N57; PF74 makes further interactions with Q63 and K70, while BI-2 makes only one additional interaction with the N74 side chain.

More recently, second-generation capsid-binding drugs have been developed, first GS-CA1 (Yant et al., 2019) and then GS-6207 (Lenacapavir). Similar to PF74 and BI-2, Lenacapavir also interacts with the FG-binding pocket but has been shown to be much more potent with sub-nanomolar inhibition (Bester et al., 2020; Link et al., 2020), contrasting with the micromolar inhibition observed with PF74 (Shi et al., 2011; Price et al., 2014). Lenacapavir is also currently being investigated in clinical trials as a long-acting antiretroviral (Segal-Maurer et al., 2022; Gupta et al., 2023). Lenacapavir is a much larger compound than either PF74 or BI-2, with a molecular weight approaching 1 kDa (Figure 3C). Examination of the Lenacapavir-binding site reveals how this larger molecule buries ∼800 Å^2^ at the drug–protein interface and exploits a much greater proportion of the available residues in the FG pocket that interact with the natural ligands and the first-generation compounds. Specifically, Lenacapavir packs against the hydrophobic residues projecting from helices α3 and α4, making interactions across the whole FG pocket utilising both fluorine interactions with capsid residues as well as hydrogen bonding with the N57, K70, and N74 side chains. Moreover, additional interactions with Q179 in the CTD and S41 in the NTD of the adjacent capsid molecule further stabilises the interface (Figure 3C). In this way, Lenacapavir utilises nearly all the available chemical space around the FG pocket, resulting in the sub-nanomolar binding and enhanced antiviral properties over the first-generation compounds.

## Anti-capsid drugs and their mode of action

Several groups have reported that capsid-binding drugs have differing phenotypic effects depending on the concentration used. At concentrations >10 µM, PF74 and Coumermycin-A1 have been shown to accelerate core uncoating and, at least for PF74, inhibit reverse transcription. However, at lower concentrations, PF74 also blocks infection, which can be overcome by washout of the drug. This has led to the postulate that, at low doses, PF74 and BI-2 might compete at the FG pocket with natural capsid-binding ligands that are required for productive infection, such as CPSF6 and/or Nup153 (Price et al., 2014). At higher doses, PF74 is proposed to induce structural changes that rigidify the capsid lattice and allow the loss of capsid content (Marquez et al., 2018). These structural changes may cause irreversible inhibition of reverse transcription. Similarly, the inhibition profiles of GS-CA1 and Lenacapavir are also complex, with low doses of ∼1 nM mainly affecting integration and nuclear import, potentially through competition at the FG pocket, but at higher doses affecting reverse transcription, possibly through structural effects on the capsid (Yant et al., 2019). Understanding how sub-nanomolar amounts of Lenacapavir compete with FG-binding host factors is likely to be heavily researched in the future. One possibility is that significant amounts of FG-binding host factors need to interact with the HIV-1 capsid to promote migration across the nuclear pore or to target the capsid-containing PIC to the chromatin integration site. Therefore, even if Lenacapavir only blocks a fraction of binding sites, it still prevents enough CPSF6 or Nup153 from binding to function efficiently. On the other hand, given that Lenacapavir has been proposed to stabilise the capsid, it is also possible that binding of Lenacapavir induces conformational allosteric effects on the capsid that prevent productive binding of host factors at sites distal to those bound by Lenacapavir. Whatever is the case, it is apparent that the revolution brought about by the discovery and production of second-generation capsid-binding molecules will have long-lasting beneficial consequences both in the clinical setting and for the advancement of our fundamental understanding of the early events in HIV-1 replication.
